# The partial clinical remission phase of type 1 diabetes: early-onset dyslipidemia, long-term complications, and disease-modifying therapies

**DOI:** 10.3389/fendo.2025.1462249

**Published:** 2025-04-16

**Authors:** Benjamin Udoka Nwosu

**Affiliations:** ^1^ Division of Endocrinology, Department of Pediatrics, Zucker School of Medicine at Hofstra/Northwell, New York, NY, United States; ^2^ Division of Endocrinology, Department of Pediatrics, University of Massachusetts Medical School, Worcester, MA, United States

**Keywords:** type 1 diabetes, type 2 diabetes, dyslipidemia, hyperglycemic memory, hyperlipidemic memory, honeymoon phase, partial clinical remission

## Abstract

No therapy confers complete β-cell protection at any of the 3 stages of type 1 diabetes (T1D). Disease-modifying therapies in type 1 diabetes aim to prolong the preclinical (stages I and II) and the post-diagnostic partial clinical remission (PR) phases of T1D to reduce its short- and long-term complications. These therapies are focused on mitigating β-cell apoptosis by reducing autoimmune attacks on surviving β-cells through several pathways; as well as improving β-cell function to enable the production of functional endogenous insulin and C-peptide through the reduction of proinsulin to C-peptide ratios and other measures. These therapies target the 3 stages of T1D as monotherapy or combination therapy. Stage I of T1D is marked by the presence of at least one diabetes-associated autoantibody in an individual with normoglycemia; stage II is marked by the presence of diabetes-associated autoantibodies and dysglycemia; stage III is marked by the clinical diagnosis of T1D in an individual with antibodies, hyperglycemia, and symptoms. Conventional thinking suggests that the long-term complications of diabetes are principally rooted in early-stage hyperglycemia at the time of diagnosis of the disease, i.e., stage III of T1D. However, this theory of hyperglycemic memory is limited as it does not address the dichotomy in lipid-based atherosclerotic cardiovascular disease (ASCVD) risk in those with T1D. Given the current limitations to developing disease-modifying therapies in T1D because of the limited impact of current agents on β-cell preservation, we introduce the theory of hyperlipidemic memory of type 1 diabetes. This theory was developed by the author in 2022 using the same population as in this article to address the shortcomings of the theory of hyperglycemic memory and explain that the dichotomy in ASCVD risk is based on PR history. In this Review, the theory presents new pathways for disease-modifying therapies in T1D that focus on preventing early-phase dyslipidemia. It is hoped that including this theoretical framework in designing disease-modifying therapies in T1D will help move the field forward. This new theory supports the hypothesis that PR is an imprimatur rather than a process. It hypothesizes that pre-diagnostic interventions, at stages I or II of T1D, to ensure the occurrence of PR may be more effective in the long term than post-diagnostic interventions, at stage III, to prolong PR. This paradigm shift in approach to disease-modifying therapy in T1D is discussed in this review.

## 1 Introduction

Diabetes mellitus affects 38.4 million Americans or 11.6% of the population (1). The leading cause of death in individuals with diabetes is atherosclerotic cardiovascular disease (ASCVD) (2). The total cost of diabetes care and management in the United States in 2022 was more than $412.9 billion (3). More than 50% of patients with type 2 diabetes (T2D) have pre-existing CVD at the time of diagnosis (4). However, there are no clear data on **early** CVD prevalence in patients with type 1 diabetes (T1D) (5), despite the high mortality from coronary artery disease of approximately 3- to 10-fold higher than in the general population (4).

### 1.1 The partial clinical remission phase of type 1 diabetes

T1D is marked by persistent hyperglycemia resulting from autoimmune destruction of the pancreatic β-cells (6). A period of partial clinical remission (PR) may follow the diagnosis of T1D, i.e., stage III of the disease, and this phase is marked by an increased functionality of the surviving β-cells with attendant endogenous insulin production (7, 8). Subjects who experienced PR are designated as remitters and those who did not are designated as non-remitters. PR typically lasts for 3-12 months (9), but could extend for decades into the established phase of T1D (10).

Despite the strong correlation between ASCVD and diabetes mellitus, the underlying mechanisms remain poorly understood (11), especially in T1D where 50% of the subjects undergo PR or honeymoon phase following the diagnosis of T1D (7, 12–14). However, the impact of PR on the earliest lipid phenotypes in individuals with T1D is not fully understood (15). Though PR has been reported to modulate the degree of early-phase dyslipidemia (16), mid-term microvascular disease risk (12), and long-term ASCVD risk (17), only one adult study (15) has directly compared the earliest phenotype of lipid-based ASCVD risk between subjects with T2D and T1D, after stratifying the subjects with T1D into remitters and non-remitters based on their PR history. Such stratifications are important to establish the nature and prevalence of dyslipidemia in T1D. PR-based stratified studies in patients with T1D will help to clarify unexamined contributors to diabetic dyslipidemia in children and adults with diabetes mellitus, such as the role of hyperlipidemic memory on post-diagnostic lipid phenotypes.

The Diabetes Control and Complications Trial demonstrated a protective role for C-peptide on the vasculature in remitters or patients with T1D who had residual β-cell function (17). The Medalist study (18) found that some adult patients with T1D for >50 years who were still producing endogenous insulin had better glycemic control and lipid profile compared to their peers. The T1D Exchange study (19) of 919 individuals reported that a great proportion of children and adult patients were still producing insulin several years after their diagnosis of T1D, i.e., at stage IV of T1D. This study reported the presence of residual C-peptide 3-5 years after the diagnosis of T1D in 78% of participants who were diagnosed at >18 years and 46% of those diagnosed at <18 years. They also found that 6% of subjects with childhood-onset-, and 16% of those with adult-onset T1D had residual C-peptide at 40 years or more following their diagnosis. This body of work and others form the basis for the current disease-modifying therapies in T1D to protect the β-cells, augment β-cells function, or expand the β-cell mass, **Table 1**.

**Table 1:** Key disease-modifying therapies for type 1 diabetes that have been tested in clinical trials.

Disease Modifying Agent	Mechanisms	Effect on C-peptide_AUC and proinsulin-to-C-peptide (PI:C) ratio vs. placebo	Metabolic impact on glycemia and total daily dose of insulin (TDDI)	Safety concerns
Vitamin D Nwosu 2022 (20) Nwosu 2024 (21)	β-cell protection via reduction in β-cell stress through (a) reduced proinsulin to C-peptide ratio, and (b) tumor necrosis factor-alpha	• 20% higher C_AUC at 52 weeks, with an improved effect with time (N=36). • Reduction in PI:C	Vitamin D significantly reduced the temporal trends in A1c. No difference in the TDDI	Excellent safety profile.
Vitamin D and saxagliptin Yan et al. (22)	A combination of vitamin D’s mechanism of action as noted above and a dipeptidyl peptidase 4 (DPP-4) inhibitor’s role in preventing the degradation of glucagon-like peptide-1 which is associated with improved β-cell function	• The combination slowed the decrease in C_AUC at 24 months (N=301). No effect of saxaglipitin alone. • No data on PI:C	No difference in A1c. Significant reduction in the TDDI in the combination group.	No increased risk as monotherapy or combination therapy
Liraglutide (the New Lira Study) Dejgaard et al. (23)	Improved β-cell function by a GLP-1 receptor agonist	• Significantly higher AUC C-peptide in the Liraglutide vs placebo group: 176, 95 CI 142-208 nmol/L vs 120; 95 CI 97-143 nmol/L (N=68) • No data on PI:C	No significant difference in A1c. Significant reduction in the TDDI in the Liraglutide group	Gastrointestinal adverse effects. No increased risk for hypoglycemia
Vitamin D and Lansoprazole Reddy et al. (24)	A combination of vitamin D’s mechanism of action as noted above and lansoprazole’s proton pump inhibition which increases gastrin level and in turn increases β-cell neogenesis and survival	• No data on C_AUC • Slower reduction in fasting C-peptide at 6 months, 31% vs 48% (N=28). • No data on PI:C	No difference in A1c. Lower TDDI in the experimental group	No increased risk of adverse events.
Imatinib Gitelman 2021 (25)	Tyrosine kinase inhibitor that reduces β-cell endoplasmic reticulum stress and apoptosis	• 19% improvement at 12 months, no effect at 24 months (N=67). • No impact on PI:C	No difference in A1c or TDDI	Increased risk for side effects
Anti-IL-21 Liraglutide Trial Von Herrath 2021 (26)	A combination of the immunomodulatory activities of anti-IL21 antibody and the activities of Liraglutide, a GLP-1 receptor agonist to improve β-cell function	• Reduced C-peptide loss by combination therapy at 54 weeks, 10% vs 39% (N=308) • No impact by either anti-IL-2 or Liraglutide alone • No data on PI:C	Reduction in TDDI at 54 weeks in the combination arm versus placebo. No difference in A1c.	No increased risk.
Teplizumab Ramos 2023 (27)	Anti-CD 3 monoclonal antibody that protects the β cells by increasing the apoptosis of activated T cells while sparing regulatory T lymphocytes	• 59% higher C_AUC at 78 weeks (N=217). Effect stable over time. • No data on PI:C	No difference in A1c or TDDI	Headache, gastrointestinal symptoms, rash, lymphopenia, cytokine release syndrome
Bariticinib Waibel 2023 (28)	An oral Janus kinase (JAK) inhibitor that prevents the expression of cytokine-induced HLA-1 in islet cells and thus prevents CD8+ T cell activation	• 48% higher effect size for the median at 48 weeks (N=91). • No data on PI:C	No significant difference in A1c. Lower glucose variability and higher % time in range in the bariticinib group	No increased risk or acceptable side effect profile
Verapamil Forlenza 2023 (29)	Reduces thioredoxin-interacting protein expression that is linked to β-cell apoptosis	• 30% higher C-peptide level at 52 weeks (N=88). Effect stable over time. • No data on PI:C	No difference in A1c or TDDI	Increased risk associated with depression, nausea, vomiting, EKG abnormalities
REPAIR-T1D Trial: sitagliptin and lansoprazole Griffin et al. (30)	A combination of a dipeptidyl peptidase 4 (DPP-4) inhibitor’s role in preventing the degradation of glucagon-like peptide-1 which is associated with improved β-cell function, and lansoprazole’s proton pump inhibition which increases gastrin level and in turn increases β-cell neogenesis and survival	• No change (N=68). • No data on PI:C	No significant difference in metabolic outcomes between the groups	No increased risk
Low-dose anti-thymocyte globulin (ATG) and pegylated granulocyte colony-stimulating factor (GCSF) Haller et al. (31)	A combination of the immunomodulatory properties of ATG and GCSF to preserve residual β-cell function	• 40-50% effect size at 12 months (N=89) • Significantly higher AUC C-peptide in ATG cohort vs placebo, 0.646 nmol/L vs 0.406 nmol/L. • No difference in AUC C-peptide in those treated with combination ATG + GCSF • No data on PI:C	Lower A1c by ATG and ATG+GCSF versus placebo but no significant difference in the TDDI	Serum sickness, cytokine release syndrome, lymphopenia, musculoskeletal and connective tissue complaints

Despite these landmark findings, there is a paucity of data on the characterization of early-onset, post-diagnostic lipid phenotypes in remitters and non-remitters (16) across the lifespan in both children and adults to enable the translation of crucial clinical data to PR-based ASCVD clinical guidelines. For example, there is currently no consensus on dyslipidemia in children and adolescents with T1D, as studies have reached differing conclusions, and it is believed that a lack of stratification of subjects by PR history may have confounded these results (32–35). Similarly, the literature in adults with diabetes mellitus showed that while the risk factors for ASCVD are well established in T2D (36), they are unclear in those with T1D (5, 11). In general, the lack of an understanding of the degree of PR-based dichotomy in early lipid phenotypes in subjects with T1D, i.e., remitters and non-remitters, and the assumption that subjects with T2D have worse lipid profiles than those with T1D have hindered a thorough assessment of the intrinsic differences in lipid phenotypes in patients with T1D (36) (5, 11),. As a result, the risk factors for ASCVD are well established in individuals with T2D (36), but not in those with T1D (5, 11).

Current knowledge indicates that several factors such as HbA1c concentration, diabetic nephropathy, hypertension, and dyslipidemia are important risk factors for ASCVD in adults with established T1D (37). However, the phenotype of the earliest ASCVD risk profile at the time of diagnosis of T1D, i.e., stage III of T1D, compared to T2D, and the cardinal role of PR on early lipid phenotype in those with T1D, which presages later ASCVD risk status, are not fully characterized.

This review article aims to address this important gap in knowledge with an emphasis on how this new lipid-based paradigm could be applied to pharmacologic interventions to augment the PR of T1D.

### 1.2 Prevalence of the partial clinical remission phase of type 1 diabetes

The introduction of the gold-standard clinical definition for PR, the insulin-dose adjusted hemoglobin A1c (IDAA1c) in 2009, has enabled a consensus on the estimation of PR in clinical practice (9). Recent studies show that the prevalence of PR in children and adolescents is approximately 50% (7, 12–14). This suggests that PR does not occur in a significant proportion of children and adolescents diagnosed with T1D (38–41). These individuals are referred to as non-remitters.

This high proportion of non-remitters reflects a key deficiency in the early management of children and adolescents T1D as there are no guidelines to prevent or address the early-onset dyslipidemia that occur in non-remitters (7, 12, 41, 42).

Despite increasing reports showing that remitters have a significant long-term prognostic advantage over non-remitters, this dichotomy in risk has not been considered in the early phase of diabetes management, as there is no clear guidance on strategies to prevent early-onset dyslipidemia in non-remitters, which represents a key drawback in early management T1D in children (7, 12, 41, 42). Additionally, the fact that approximately 50% of children with T1D will not experience PR suggests that post-diagnostic interventions at stage III of T1D might not be very useful in this subset of patients with T1D, i.e., the non-remitters.

### 1.3 The mechanism of the partial clinical remission phase of type 1 diabetes and associated theories of remission

#### 1.3.1 The mechanisms of non-remission

The molecular mechanisms that determine the occurrence of remission or non-remission are not fully characterized (43). However, certain key factors have been identified. These include increased β-cell stress as marked by increased proinsulin-to-C-peptide (PI:C) ratio (44), increased glucagon concentration (45), unfavorable cytokine profile (46), and the role of immune mediators and genetic markers (43). Available data show that remitters possess a distinctive cytokine profile that protects the β-cells (46). Glucagon concentration is lower in remitters, which supports the premise that glucagon production is suppressed by intra-islet insulin production and release (45). Data supporting the key role for immune mechanisms in PR (47) show significantly lower concentrations of interferon-γ in remitters compared to non-remitters and controls, a higher frequency of CD4^+^ CD25^+^-CD127^hi^ cells, and a non-Treg subset of memory T cell, which are all consistent with a slower rate of progression of T1D (48, 49). This supports the hypothesis that immune mediators could protect the β-cells and thus prolong PR. Moya et al. (49) suggested that the duration of PR could be predicted using a combination of the frequency of the CD4^+^ CD25^+^-CD127^hi^ cells with glycemic markers at the time of diagnosis of T1D. In new-onset T1D, elevated islet antigen-specific interleukin-10-producing cells correlate with improved glycemia, while increased FoxP3 expression predicts a worse outcome (50). A genetic study (51) of patients with newly diagnosed T1D found that the level of circulating microRNA, has-miR-197-3p, at 3 months after T1D diagnosis strongly predicted the magnitude of residual β-cell function one year after the diagnosis of T1D. The marker for increased β-cell strain is poor proinsulin processing as data indicate that individuals who are more likely to undergo remission, such as overweight male children, have efficient proinsulin processing (44). Children with new-onset T1D generally have elevated PI:C ratio (52, 53). However, Nwosu et al. (21) recently demonstrated that high-dose vitamin D reduces PI:C ratio in this population. Clinical studies show that younger age at diagnosis, female sex, severe acidosis, and increased numbers of diabetes-associated autoantibodies are associated with non-remission (54). Thus, the occurrence and the duration of remission, or non-remission is determined by a constellation of genetic, immune, hormonal, and inflammatory factors.

## 2 Proposed theories to explain the partial clinical remission phase of type 1 diabetes

### 2.1 Impact of optimal glycemia on residual β-cell function

Data from the landmark Diabetes Control and Complications Trial suggested that intensive glycemic control following the diagnosis of T1D could preserve RBCF (17, 55). However, recent investigations in this area have shown mixed results, whereas some studies provide support for the DCCT findings (56), other studies (57–59) and systematic reviews (60) failed to show that improved glycemic control prolongs RBCF in subjects with new-onset T1D. A study by Enander et al. (59) is particularly interesting as it showed that RBCF at 2 years was associated with the initial A1c and C-peptide concentrations, but was independent of initial insulin regimens. This suggests that PR is rather a unique event or an imprint in the life history of T1D that occurs at the time of diagnosis of T1D and is determined by the constellation of prevailing factors at the time, i.e., stage III of T1D, such as the degrees of glucotoxicity and lipotoxicity (43). This suggests that interventions to prolong RBCF should focus on key pre-diagnostic pathways at stages I and II of T1D to either reduce β-cell stress through the use of agents such as high-dose vitamin D to reduce the PI:C ratio (21), or immunomodulators such as teplizumab to reduce the impact of autoreactive T cells on β-cells (61), and not necessarily on interventions at stage III of T1D to alter the post-diagnostic glycemia. This new paradigm that RBCF is independent of post-diagnosis glycemia at stage III is supported by the theory of hyperlipidemic memory (62) and the concept of PR *imprimatur* where improved diabetes outcomes are largely independent of post-diagnosis glycemia, but rather on the dichotomy in lipid phenotypes that is determined by PR history (62).

We now examine the theories of hyperglycemic memory and hyperlipidemic memory in detail.

### 2.2 The theory of hyperglycemic memory of type 1 diabetes

Landmark studies in T1D show that intensive glycemic management preserves RBCF following the diagnosis of T1D (17, 55). The occurrence of residual endogenous insulin secretion in patients with T1D has been linked to reduced risk for severe hypoglycemia (63, 64), reduced development of diabetic retinopathy (65), promotion of statural growth in prepubertal children (66) and reduced risk for long-term complications of T1D (12, 17).

In contrast, the non-remitters experience chronic hyperglycemia from the time of diagnosis (12, 15). This initial phase of chronic hyperglycemia has been associated with long-term complications of diabetes mellitus, regardless of whether glycemia improved later in the history of the disease (43, 59). This phenomenology of diabetes complications arising from an initial chronic hyperglycemia has been christened the theory of hyperglycemic memory (67). Recent studies indicate that there are non-glycemic contributors to the phenomenon of hyperglycemic memory, and most of these factors are not fully characterized (43). As a result, some investigators now refer to this phenomenon as the glyco-metabolic theory (43). The researchers suggest that the mechanisms leading to glyco-metabolic memory are *interdependent* and act simultaneously. The 4 proposed mechanisms are oxidative stress, generation of advanced glycation end-products, chronic inflammation, and epigenetic changes (43). However, these studies did not examine the initial post-diagnostic lipid phenotypes in patients with newly diagnosed T1D to determine whether a dichotomy exists in the lipid parameters (between remitters and non-remitters) and whether non-remission is associated with both hyperglycemia and hyperlipidemia. Therefore, the theory of hyperglycemic memory has limited application as it does not explain the glycemia-independent dichotomy in early lipid phenotypes that presages subsequent divergence in ASCVD risks in patients with T1D.

### 2.3 The theory of hyperlipidemic memory of type 1 diabetes

**Figure 1:**
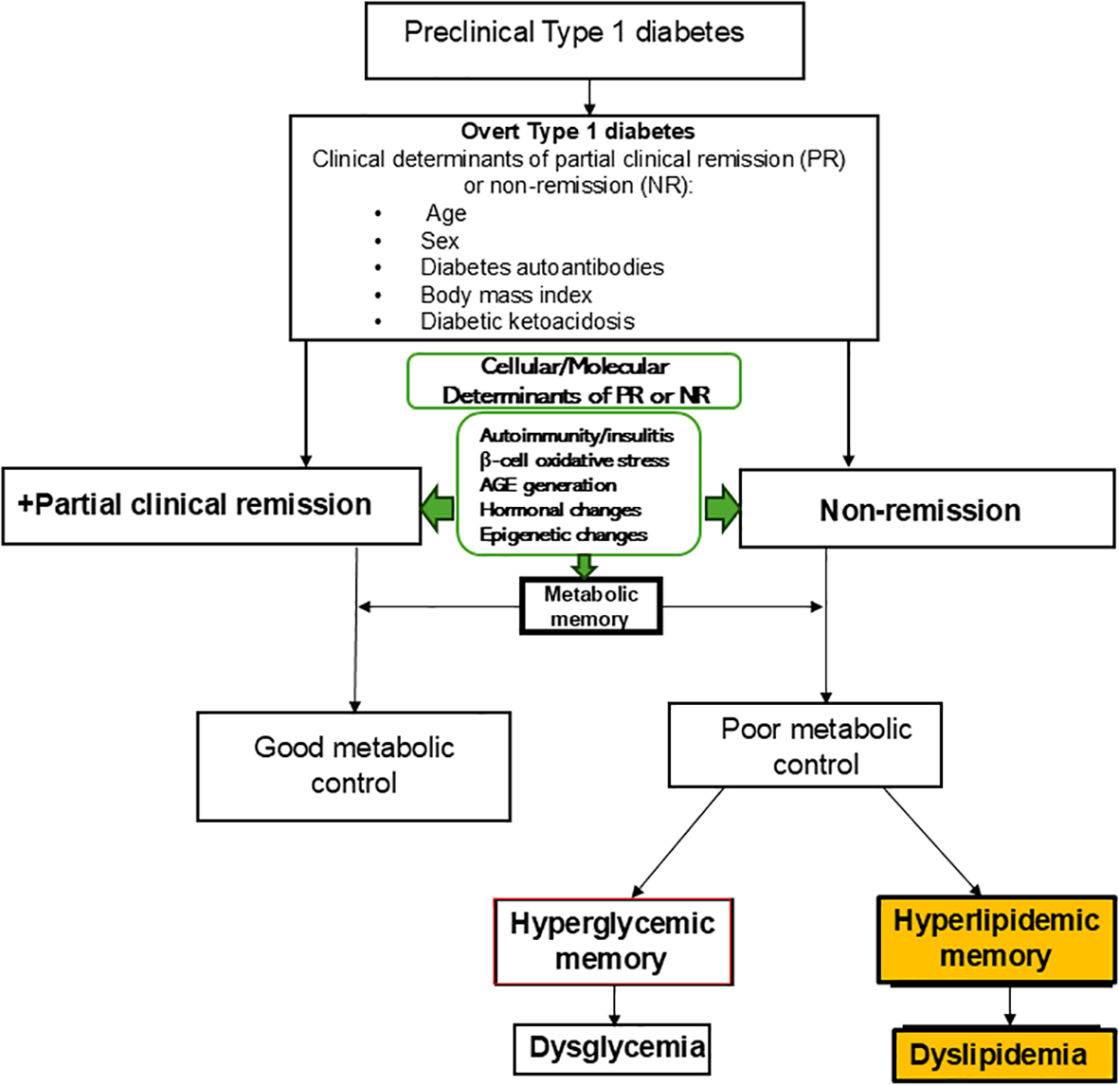
The theory of hyperlipidemic memory of type 1 diabetes explains the early dichotomy in lipid parameters in patients with type 1 diabetes (T1D) and the attendant divergence in dyslipidemic atherosclerotic cardiovascular disease risk in non-remitters compared to the remitters. The theory of hyperglycemic memory explains dysglycemia in non-remitters while the theory of hyperlipidemic memory explains dyslipidemia in non-remitters. This model shows the relationships between the overt clinical determinants of remission or non-remission and the less overt molecular and cellular factors that also predict the occurrence of remission or non-remission. These clinical, molecular, and cellular determinants impact metabolic memory and thus the long-term trajectory of the clinical course and complications of T1D. The concept of PR imprimatur calls for disease-modifying interventions in the preclinical stages of T1D to ensure robust metabolic memory and decreased long-term complications of T1D.

As a result of the shortcomings of the theory of hyperglycemic memory, Nwosu (62) proposed the theory of *hyperlipidemic* memory of T1D which explains the divergence in lipid-based ASCVD risk and provides the necessary framework to understand the differences in lipid phenotypes between remitters and non-remitters on one hand, and between those with newly-diagnosed T1D or type 2 diabetes (T2D) on the other (**Figure 1**).

The theory of *hyperlipidemic* memory of T1D is premised on five years of research on the early post-diagnostic dichotomy in lipid phenotypes between remitters and non-remitters across the lifespan in children, adolescents, and adult patients with newly diagnosed T1D, T2D, and matched controls. This theory provides a rigorous explanation for the differences in lifelong ASCVD risk between remitters and non-remitters.

Nwosu and his colleagues (62) developed this theory by conducting 4 clinical studies involving children, adolescents, and adults to characterize the features of hyperlipidemic memory. In the first investigation (16), they explored the impact of the presence or absence of PR on lipid parameters in youth five years after their diagnosis of T1D.

In the second study (68), they investigated whether pubertal maturation influenced the dichotomy in lipid profiles in T1D; and whether pubertal lipid dichotomy occurred in age-matched healthy youth without a diagnosis of T1D.

In the third study (69), they used the findings from patients with T1D and control subjects to investigate early lipid changes in T2D by comparing the earliest lipid phenotypes of subjects with T2D to those of remitters, non-remitters, and controls. Finally, in the fourth study (15), they examined the impact of PR on the earliest lipid phenotypes in adult subjects with either T1D or T2D, and their matched controls.

In these 4 studies, the investigators found that remission was more robust in male than female subjects; and that remitters had significantly favorable lipid profiles compared to non-remitters (16) (68) (69) (15) as shown by significantly lower LDL-cholesterol, non-HDL cholesterol, total cholesterol, TC/HDL ratio, and mean composite scores for lipid-based ASCVD risks in the remitters. The early dyslipidemia in non-remitters was similar to that of the obese patients or those with T2D, while the favorable lipid profile in the remitters was similar to that of the normal-weight controls. These findings are similar to the results of a longitudinal study that reported a significantly reduced risk for chronic microvascular complications at 7-year follow-up in young adults who experienced PR (12).

This body of work across the lifespan in children, adolescents, and adults supports the theory of hyperlipidemic memory. This new theory clarifies why PR largely determines the risks for early-phase dyslipidemia, mid-term microvascular disease risk, and long-term ASCVD risk in subjects with T1D.

## 3 Support for PR-mediated hyperlipidemic memory as the primary determinant of early lipid phenotypes in both pediatric and adult type 1 diabetes

It is important to analyze the risk factors for dyslipidemia such as glycemia, BMI, and insulin resistance in patients with either T1D or T2D to understand the key role of hyperlipidemic memory on early lipid phenotypes in T1D. Nwosu et al. (16) examined the role of early glycemia in T1D. They found that both remitters and non-remitters have hyperglycemia at the time of diagnosis of T1D, but that glycemia improves markedly in the remitters and less so in the non-remitters, suggesting that hyperglycemia from poor glucose management could lead to dyslipidemia in these patients. However, they noted in their follow-up study that included patients with T2D (69), who had unfavorable lipid parameters at the time of the diagnosis, but with significantly lower mean A1c level of 6.7% compared to the mean A1c levels of the T1D cohort (8.8% for the non-remitters, and 8.6% for the remitters). These findings argue against hyperglycemia as the key determinant of early-phase dyslipidemia in children with either T1D or T2D. These data and previous reports (43, 59) show the limitations of the theory of *hyperglycemic* memory to explain the PR-mediated divergence in lipid phenotypes in patients with T1D.

Furthermore, though BMI predicts dyslipidemia, the occurrence of normal BMI z-scores in the non-remitters with a BMI z-score of 0.63 ± 0.9, despite having a similar lipid profile as the obese patients with T2D with a BMI z-score of 2.4 ± 0.4, suggests that increased BMI alone does not explain the increased dyslipidemia in the early phases of T1D in children. This is supported by an analysis of the proportion of subjects with dyslipidemia in that study (70) that showed that LDL-C of >130 mg/dL occurred in 7 (13.2%) of the subjects with T2D; 6 (7.6%) non-remitters; 2 (4.6%) remitters; and 4 (5.5%) controls. Additionally, TC of >200 mg/dL occurred in 15 (28.3%) of the subjects with T2D; 9 (11.4%) non-remitters; 3 (6.8%) remitters; and 4 (5.5%) controls. This analysis suggests that the non-remitters and the subjects with T2D, despite their differences in BMI z-scores, had a *higher frequency of dyslipidemia* compared to the remitters and controls.

Finally, the similarity of early lipid profiles in patients with T2D and the non-remitters, despite their significant differences in BMI z scores, also argues against IR as the primary determinant of dyslipidemia in non-remitters compared to the subjects with T2D. Taken together, these findings (15) establish PR as the principal determinant of early lipid phenotypes, and the divergence in ASCVD risks in both pediatric and adult patients with T1D.

## 4 Conclusions and future directions on disease-modifying therapies in type 1 diabetes to limit atherosclerotic cardiovascular disease risk

Partial clinical remission (PR) is a key event in the life history of T1D. When patients are stratified by PR status into remitters and non-remitters, the non-remitters have less favorable lipid phenotypes than the remitters and controls. These findings support a dichotomy in ASCVD risk in subjects with T1D that favors the remitters. This divergence in ASCVD risk is explained by the theory of hyperlipidemic memory where the initial, early-onset hyperlipidemia in non-remitters persists across the lifespan leading to increased risk for ASCVD in this sub-population of subjects with T1D. In contrast, the imprimatur of PR in the remitters presages a lifetime of favorable lipid profile which has been confirmed in large studies (10, 17). The concept of PR imprimatur is fitting because the metabolic advantages of PR continue long after the end of partial remission (12, 18). This theory of hyperlipidemic memory explains the principal role of PR occurrence or *imprimatur* on the early dichotomy in lipid phenotypes in T1D, and the subsequent divergence in lipid-based ASCVD risks.

Therefore, we propose that the advantages conferred by PR occur at the time of the diagnosis of T1D, i.e., stage III of T1D, as an imprimatur, and not as a process that follows the diagnosis of the disease. Thus, the advantages of PR need not necessarily depend on the duration of PR, but on its singular occurrence, as it encompasses a constellation of factors that protects the β-cells from the initial shock of the diagnosis of T1D that resets the long-term trajectory of the complications of the disease in a favorable path.

This new paradigm provides a new structure for early and accurate quantification of ASCVD risk in subjects with T1D across the lifespan which may lead to the development of disease-modifying agents to address the risk for early-stage dyslipidemia at stages I and II before the diagnosis of T1D using high-risk population screening. This new model calls for the inclusion of lipid-based pathways in devising strategies and therapies to either augment PR following the diagnosis of T1D at stage III or develop agents or therapies to protect the β-cells prior to the diagnosis of T1D at stages I and II in individuals at high risk of the disease. For example, proinsulin to C-peptide ratio, a marker of β-cells endoplasmic reticulum stress, is increased in children and adolescents with new-onset T1D (53). Thus, agents that reduce PI:C ratio, such as high-dose vitamin D (21) could be used at stages I and II to protect the β-cells in individuals at high risk for developing T1D to ensure that these patients experience PR and the advantages of PR to reduce the short- and long-term complications of T1D.
